# Integration of antenatal care services with health programmes in low– and middle–income countries: systematic review

**DOI:** 10.7189/jogh.06.010403

**Published:** 2016-06

**Authors:** Thyra E de Jongh, Ipek Gurol–Urganci, Elizabeth Allen, Nina Jiayue Zhu, Rifat Atun

**Affiliations:** 1 Technopolis Group, Amsterdam, the Netherlands; 2 London School of Hygiene and Tropical Medicine, London, UK; 3 Department of Global Health and Population, Harvard T.H. Chan School of Public Health, Harvard University, Boston MA, USA

## Abstract

**Background:**

Antenatal care (ANC) presents a potentially valuable platform for integrated delivery of additional health services for pregnant women–services that are vital to reduce the persistently high rates of maternal and neonatal mortality in low– and middle–income countries (LMICs). However, there is limited evidence on the impact of integrating health services with ANC to guide policy. This review assesses the impact of integration of postnatal and other health services with ANC on health services uptake and utilisation, health outcomes and user experience of care in LMICs.

**Methods:**

Cochrane Library, MEDLINE, Embase, CINAHL Plus, POPLINE and Global Health were searched for studies that compared integrated models for delivery of postnatal and other health services with ANC to non–integrated models. Risk of bias of included studies was assessed using the Cochrane Effective Practice and Organisation of Care (EPOC) criteria and the Newcastle–Ottawa Scale, depending on the study design. Due to high heterogeneity no meta–analysis could be conducted. Results are presented narratively.

**Findings:**

12 studies were included in the review. Limited evidence, with moderate– to high–risk of bias, suggests that integrated service delivery results in improved uptake of essential health services for women, earlier initiation of treatment, and better health outcomes. Women also reported improved satisfaction with integrated services.

**Conclusions:**

The reported evidence is largely based on non–randomised studies with poor generalizability, and therefore offers very limited policy guidance. More rigorously conducted and geographically diverse studies are needed to better ascertain and quantify the health and economic benefits of integrating health services with ANC.

Since 2005, antenatal care (ANC) coverage has risen considerably worldwide [1]. The World Health Organization (WHO) estimates suggest that during 2005–2012 approximately 80.5% of pregnant women globally, including 71.8% of women in low–income countries, had at least one ANC visit during pregnancy [1]. ANC provides an opportunity for women to access effective interventions that reduce risks associated with pregnancy and improve their health and well–being, as well as that of their progeny. However, while there was considerable progress towards the Millennium Development Goals 4 (to reduce child mortality) and 5 (to improve maternal health), maternal and neonatal mortality from preventable pregnancy– and birth–related complications remain high, particularly in low– and middle–income countries (LMICs) [2]. In 2013, around 289 000 women died during and following pregnancy and childbirth–the vast majority in low–resource settings [3]. Between one–third and one–half of these pregnancy–related deaths are due to preventable complications, such as eclampsia and haemorrhage, directly related to inadequate care [4]. Additionally, nearly three million newborns died during their first month of life, in large part due to insufficient provision of postnatal care (PNC) [2,5–8]. Lack of PNC not only affects neonatal mortality, but also has long–term negative impacts on the development of children who survive, as opportunities for promoting healthy home behaviours are missed. The unacceptably high maternal and neonatal mortality rates in LMICs suggest new approaches are needed to expand access to ANC, improve the quality of services provided during ANC contact, and strengthen continuity and quality of care through to the postnatal period.

In most LMICs pregnancy often marks a woman’s first encounter with formal health services, and ANC can serve as an effective platform for a broad range of health interventions [9], including for the provision of services for conditions that increase the risk of complications during pregnancy (eg, malaria, sexually transmitted infections (STIs), HIV/AIDS, tuberculosis (TB), tetanus, and malnutrition). Integrating ANC with malaria, STIs, HIV/AIDS and TB services can also expand the reach of these programmes to a broader population [10]. In settings where the prevalence of such conditions is high, integrating ANC with cost–effective services like prevention of mother to child transmission (PMTCT) of HIV [11], intermittent preventative treatment in pregnancy for malaria, and provision of insecticide treated nets [9] would likely improve maternal and child health outcomes. The WHO has identified integration of ANC with other health services, including PNC, as a key strategy for reducing missed opportunities for patient contact and for effectively and comprehensively addressing the health and social needs of pregnant women and their children, thereby improving maternal and child health [5,8,9].

Integration in health systems is variously defined [12–15], referring to establishing joint systems for organisation, financing, management, planning and evaluation of health programmes at different levels of the health system (from health facilities to ministry of health level) to improve the efficiency and effectiveness of health systems [16]. Integrated care has also been defined by WHO as “bringing together inputs, delivery, management and organization of services related to diagnosis, treatment, care, rehabilitation and health promotion” in order to “improve services in relation to access, quality, user satisfaction and efficiency” [17,18]. The rationale for integrating health services is to improve user access to health services across the care continuum to meet users’ health needs over time [19,20] and to create positive synergies among investments in health programmes [21].

However, ‘injudicious integration’ may also have harmful consequences for already constrained health systems [22]. For example, provision of multiple services during a single point of contact requires that health care providers be sufficiently trained in all aspects of the services concerned to ensure high quality care. But, in resource constrained systems training can take away health staff from frontline services [23]. Furthermore, provision of multiple services could stretch the already limited capacity, thus leading to long waiting times and hindering access for women who have to travel far to reach health facilities. In an attempt to reduce workload providers may reduce the time spent on consultations, thus compromising service quality.

To date few studies have systematically examined how integration of ANC with other services could influence health outcomes, service access, efficiency, or patient satisfaction [19,24–26]. Evidence to guide policy on the best ways to integrate ANC with PNC and other health services for pregnant women and integration impact is limited. This review examines the evidence on how integration of ANC services with PNC or other health services in LMICs affects health outcomes for women and children, health care provision (including processes, outputs, service quality) and costs. The review analyses ways in which the quality of ANC can be improved through integration with PNC and other health services. Specifically, the review focuses on the impact of integrated provision of ANC services, which can take different forms, such as co–location of ANC and PNC or other health services with a single point of access, through a well–connected referral system [27,28], or by merger of services within or across a domain of care [29].

## METHODS

### Criteria for considering studies for this review

We followed Cochrane guidelines for systematic reviews [30] and included both randomised controlled trials (RCT), where randomisation could be at individual or cluster level, and non–randomised studies (NRS). Non–randomised studies are defined in the Cochrane Handbook as quantitative studies that do not use randomisation to allocate units to comparison groups, but where allocation occurs in the course of usual treatment decisions or peoples’ choices [30]. The NRS that were eligible for inclusion in this review were non–randomised controlled trials (NRCT), controlled before and after studies (CBA), interrupted time series analyses (ITS), historically controlled studies, cohort and case–control studies.

### Type of participants

We included studies focusing on pregnant women of all ages utilizing ANC services in LMICs.

### Type of interventions

We considered any study that described a change from ‘routine practice’ with the intention to integrate provision of ANC services with i) PNC or ii) other health services. Integrated service provision models included:

• Co–location of services, using a single point of access;

• Collaboration between different service providers involved in a woman’s care (eg, in integrated care teams);

• A well–organised referral system, with follow–up and feedback among different service providers.

We considered strategies promoting horizontal integration (ie, linking services at the same level of care domain), as well as vertical integration (ie, linking services across different levels of care) [29]. For inclusion, however, studies had to compare outcomes of the intervention against a control situation in which a similar set of services was delivered in a non–integrated way (ie, additional services were available to pregnant women, but were not routinely integrated into ANC).

### Type of outcome measures

We explored the impact of ANC integration on health outcomes (including health behaviour and health status for mother and child, and user experience, such as user satisfaction) as well as health care outputs (including utilisation of services, access, coverage, quality, efficiency and cost) for all relevant users and providers, and including any adverse outcomes.

### Search methods for identification of studies

We searched the Cochrane Central Register of Controlled Trials (CENTRAL), Cochrane Database of Systematic Reviews (Cochrane Reviews), Cochrane Database of Abstracts of Reviews of Effects (Other Reviews), MEDLINE (Ovid), Embase (Ovid), CINAHL Plus (EBSCO), Global Health (Ovid) and POPLINE on January 21, 2014. We used a comprehensive search strategy with no language or publication date restrictions. The search string for MEDLINE, which was tailored to each of the databases, is provided in **Online Supplementary Document(Online Supplementary Document)**. The “integration” block was adapted from the search strings used in the Cochrane EPOC review of integration of PHC services [31] and the “LMIC” block was adapted and expanded from the Medline LMIC filter.

We checked the reference lists of all included studies and examined the bibliographies of relevant systematic reviews and meta–analyses identified during the search.

### Data collection and analysis

We performed the selection of potentially eligible studies through a staged process. At every stage of the process, two authors independently assessed publications for their relevance and adherence to inclusion criteria. TdJ, EA and IGU first piloted and refined the selection process in a random sample of 100 studies to ensure high inter–rater agreement. In the first stage, the authors (TdJ, EA) evaluated publications for their potential relevance based on titles. Any title judged as potentially relevant by either of the authors was next assessed for eligibility on the basis of the abstract. All abstracts considered potentially eligible by both authors were retained for further scrutiny. Due to the large number of abstracts, those on which the authors disagreed were independently reviewed by a third author (IGU) who decided on its inclusion into the final round of screening. When no abstract was available, the publication was also retained in the selection until the full text was acquired and screened. In the final stage of screening, two authors (TdJ, EA) reviewed the full text of each retained publication to determine relevance and whether the publication met our inclusion criteria. If a study was published only as an abstract (eg, conference abstracts where full manuscript was not yet available), we only included the study if there was sufficient information presented in the abstract to demonstrate that it met the review's inclusion criteria and was of an acceptable methodological standard. In the case of disagreement between the authors, a third author (IGU) acted as an arbiter to decide upon the final inclusion.

### Data extraction and management

For studies that were deemed eligible for inclusion, we extracted the data to a standardised form including key information such as administrative data (title, author, year of publication, country, setting, funding etc.); methods (stated study design, data relevant for risk of bias assessment, duration and completeness of follow–up); and information on participants, interventions and comparisons. Quantitative results for each study were separately extracted to an Excel^TM^ spreadsheet for further analysis; and grouped by outcome measures as defined in the included studies. Two separate authors (EA, NZ) extracted the quantitative results, with independent verification by a third author (IGU).

### Assessment of risk of bias in included studies

To assess the risk of bias in the included studies, we used standardised tools appropriate to different study designs. For RCT/NRCT/cRCT/CBA we used the criteria formulated by the Cochrane Effective Practice and Organisation of Care **(**EPOC) Group, which rate each study on nine dimensions, namely: sequence generation; allocation concealment; baseline outcome measurement; baseline characteristics of participants; blinding of participants, personnel and outcome assessors; contamination; selective outcome reporting; and other sources of bias [32]. Each category was rated as low–risk, high–risk or unclear.

For cohort designs, case–control studies and historically controlled trials, we assessed risk of bias using the Newcastle–Ottawa scale, which contains only eight items and is simpler to apply than other checklists for NRS [33]. The scale uses a ‘star’ rating system with a maximum of nine stars, with ratings assigned in three categories: the selection of the study groups (four stars), the comparability of the groups (two stars) and the ascertainment of outcome of interest (three stars) (**Box 1**).

Box 1 **The Newcastle Ottawa Scale**

In the study group category one star could be awarded for each of the following 4 criteria: a) if the exposed group was representative of the average woman seeking antenatal care services and, where applicable, additional health services; b) if the control group was selected from the same community as the integrated services group, c) if the delivery of individual health services was ascertained from secure records or structured interviews, and d) if there was sufficient evidence that the outcome of interest was not present at the start of the study. In the group comparability category, one star was awarded if the study reported no significant differences in baseline characteristics. Two stars were awarded if there was statistical evidence of no baseline differences across groups or if the results were risk–adjusted (by minimum of maternal age). In the outcome category, three stars could be awarded if: a) the assessment of outcome was done by independent blind assessment or determined from secure records, b) follow–up was sufficiently long; and c) either loss to follow up was small (<5%) or if it could be sufficiently demonstrated that loss to follow–up was unlikely to have affected findings.

### Assessment of heterogeneity and data synthesis

We considered whether it was appropriate to combine the studies in a meta–analysis by investigating heterogeneity in the methodologies (eg, type of service integration, study design setting and outcomes) and results of the included studies. As there was significant heterogeneity in the included studies and the study results were not expressed using consistent effect measures, we narratively summarise the findings. We also present the results of included studies in a Forest plot, but suppressed the pooled estimate, as recommended by the Cochrane Handbook [30]. We used the Forest plot to facilitate visualisation of the results, particularly to highlight the varied quality of the evidence and heterogeneity of results.

We used odds ratios (OR) as measures of effect for dichotomous outcomes. We had planned to use standardised mean differences (SMD) for continuous outcomes and where the study reported medians, to convert the medians to means using the methods proposed in Hozo and others [34]. However, for the three studies that reported continuous outcomes, either the standard deviation for the means or the ranges for medians were missing, therefore we present continuous outcomes as reported in original studies. The analysis only used data published in the studies.

## RESULTS

**Figure 1:**
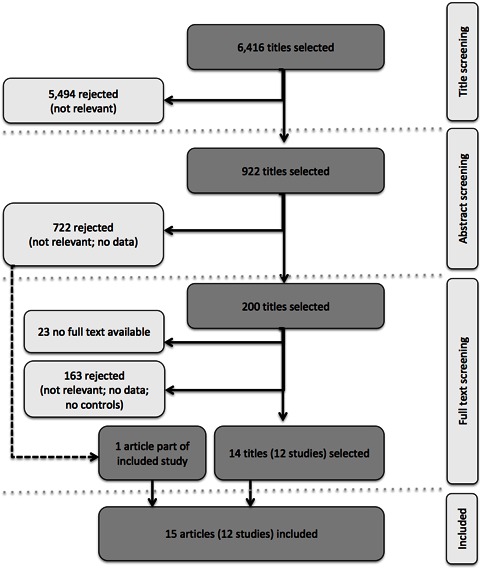
Flowchart showing process of screening and selection of studies for inclusion.

In database searches, we identified 6 416 unique citations. Of these, 922 titles were considered potentially relevant to this review. Of these citations, 842 included abstracts that were subsequently reviewed. Among the abstracts, 120 were considered potentially relevant. For an additional 80 citations no abstracts were available. These citations were all carried forward to the next stage of the screening process, in which the full text of the potentially eligible studies was reviewed. We retrieved the full text for 177 out of 200 citations. After screening against the inclusion criteria, we identified 14 citations, presenting data for 12 separate studies, that met all conditions and that were included in this review. One article that did not meet the inclusion criteria on its own was subsequently added, as it provided additional information on an already included study. The process of screening and selection is presented in **Figure 1**.

### Description of included studies

Of the 12 studies included in this review, 10 were set in Sub–Saharan Africa: three in Kenya [35–39], three in South Africa [40–43], two in Mozambique [44,45], one in Zambia [46], and one in Malawi [47]. The other two studies were set in Asia, namely in Bangladesh [48] and Mongolia [49] (**Table 1** provides a summary of the included studies). All included studies had pregnant women, either with or without their newborns, as the principal study participants. Additionally, one article described the impact of integrated services from the point of view of health care providers [37]. Excluding the latter and one other study in which the number of participants was not specified [45], the included studies represented a total of 87 755 participants, with study sizes ranging from 164 [40] to 31 526 [46] participants.

**Table 1:** Summary of included studies

**Study**	**Services integrated**	**Setting (participants)**	**Study design**	**Intervention description**	**Control description**	**Outcome measures**
**HIV**						
Geelhoed 2013 [44]	ART, PMTCT	Mozambique (376)	Controlled before–and–after study	MCH nurses provided all recommended health interventions applicable to both mother and child, including follow–up of HIV–exposed infants and early infant diagnosis of HIV, during the antenatal, postnatal, family planning, growth monitoring, high–risk child and vaccination consultations.	In the health care facilities of the control group, the same services were provided separately, one type of services after another, as is routine in the Mozambican public health care system.	Follow–up of HIV–exposed infants (registration, follow–up visits, serological testing); MCH attendance; Acceptability of integrated services to health care providers.
van’t Hoog 2005 [39]	PMTCT	Kenya (8231)	Historically controlled trial	HIV pre– and post–test counselling from an ANC nurse–counsellor; HIV testing at an on–site facility. The same counsellor also provided routine ANC preventive interventions like tetanus toxoid and sulfadoxine–pyrimethamine.	Opt–in HIV counselling was provided in a separate location within the hospital complex. HIV testing was conducted in an off–site laboratory.	Uptake of HIV counselling, testing and uptake of NVP.
Kasenga 2009 [47]	PMTCT	Malawi (1259)	Historically controlled trial	HIV testing and counselling services, and later on also management of sexually transmitted infections, were integrated within ANC.	Voluntary counselling and testing services were offered through a separate VCT unit at the outpatient department, through an opt–in approach.	Uptake of HIV testing
Killam 2010 [46]	ART	Zambia (31 536)	Stepped–wedge cluster non–randomised trial	Eligible women received ART in ANC until 6 weeks postpartum and then were referred to the general ART clinic.	Women found to be seropositive through ANC testing and eligible for ART were referred to the ART clinic, located on the same premises as ANC, but physically separated and separately staffed.	Proportion of treatment eligible pregnant women enrolling into HIV care within 60 d of HIV diagnosis; Proportion of women initiating ART during pregnancy.
Van der Merwe [40]	ART	South Africa (164)	Historically controlled trial	HIV testing, ART adherence counselling and treatment preparation took place within ANC. Thereafter, women were referred to hospital for initiation and follow–up of ARV treatment, which, whenever possible, was provided by the same staff members who began treatment preparation.	Pregnant women with indications for ARV treatment were referred to a hospital located approximately 1 km away, for preparation and initiation of treatment and long–term follow–up. These women were “fast–tracked” into treatment.	Pregnancy outcomes; Time–to–treatment initiation; Gestational age at ARV treatment initiation; Time from ARV treatment initiation to childbirth; Time between HIV diagnosis and receiving CD4 cell count results.
Ong’ech 2012 [38]	PMTCT	Kenya (363)	Prospective cohort study	Early infant HIV testing and prophylaxis were provided in the Maternal and Child Health clinic.	Infants were escorted to the Comprehensive Care Clinic, within the same health facility, for all HIV–related services.	Rates of attendance at each study visit (9 and 12 mo) and receipt of services for: infant HIV testing and prophylaxis at 6–8 weeks, receipt of immunizations at 14 weeks, continuation of prophylaxis at 6 mo, measles immunization at 9 mo, and HIV antibody testing at 12 mo.
Pfeiffer 2010 [45]	ART	Mozambique (unknown)	Retrospective cohort study	At integrated sites, HIV–positive women were referred to the ART clinic from ANC services within the same health unit.	At vertical sites, HIV–positive women were referred to the ART clinic from ANC services at other health units.	Loss to follow–up from referrals of HIV–positive women from PMTCT services to ART services.
Stitson 2010 [42]	ART	South Africa (14 987)	Retrospective cohort study	Site 1: ART initiated within the antenatal clinic when obstetricians with an HIV specialisation were on site. Site 2: women were referred by letter to a separate ART service located within 100 m of the maternity unit on the same premises.	Eligible women at the ANC clinic were referred to another site for HIV counselling and opt–in testing. ART was delivered at a separate primary health care facility approximately three kilometres from the antenatal service, using a referral letter.	Proportion of women who received more than 8 weeks of HAART; initiation of HAART in pregnancy.
Stinson 2013 [41]	ART	South Africa (14 617)	Retrospective cohort study.	See Stinson 2010.	See Stinson 2010.	Proportion of women who initiated ART before delivery; Time to treatment initiation.
Turan 2012 [36]	ART, PMTCT	Kenya (1123)	Cluster–RCT	At the fully integrated sites, HIV positive women were provided all ANC, PMTCT, and HIV services in the ANC clinic, including HAART for women who were eligible.	In the control (non–integrated) clinics ANC and basic PMTCT services were provided in one visit, with referral to a separate clinic in the same health facility for HIV care and treatment (including HAART if indicated, opportunistic infection prophylaxis, education, and adherence counselling).	Baseline data only (aims to report HIV–free infant survival at 6 mo; rates of maternal enrolment in HIV care and treatment; infant HIV testing uptake at 3 mo).
Vo 2012 [35] (substudy of Turan 2012 [36])	ART	Kenya (326)	Nested cross–sectional study	See Turan 2012	See Turan 2012	Satisfaction; Preferred service model; average wait times.
Winestone 2012 [37] (substudy to Turan 2012 [36]	ART, PMTCT	Kenya (36 providers)	Qualitative study	See Turan 2012	See Turan 2012	Provider perceptions of quality of care.
						
Munkhuu 2009 [39]	Congenital syphilis testing	Mongolia (7700)	Cluster–RCT	The one–stop service included: (i) on–site screening for syphilis using rapid syphilis tests at the first antenatal visit and at the third trimester of gestation; (ii) immediate on–site treatment for seropositive women and their sexual partners; and (iii) pre– and post–test counselling.	After being admitted to the antenatal clinic, a pregnant woman could visit any District General Hospital or the National Center of Infectious Diseases for free initial and confirmatory syphilis testing. Women testing positive would be sent to a venereologist for appropriate case management and follow–up control, including contact tracing and counselling.	Uptake of syphilis testing at the first visit and third trimester; Receipt of adequate treatment (ie, completion of 3 doses of treatment before delivery); Treatment rates for sexual partners.
Bronzan 2007 [43]	Congenital syphilis testing	South Africa (1250)	Non–randomised controlled trial	On–site antenatal syphilis screening	Off–site syphilis screening	Percentage of eligible women who received 1, 2, or 3 appropriately timed weekly doses of penicillin; Acceptability of onsite testing to nurse clinicians.
						
Rahman 2011 [48]	Various	Bangladesh (20 766)	Controlled before–and–after study	Set of maternal and neonatal interventions, following the continuum of care approach from pregnancy to delivery to the postnatal period, with improved links between community– and facility–based service delivery modes.	In the control areas, women receive pregnancy, delivery, and post–natal care from various government health facilities.	Perinatal mortality; Rates of facility deliveries and caesarean section.

ANC – Antenatal care; ART – Antiretroviral therapy; ARV – Antiretroviral; HAART – Highly active antiretroviral therapy; PMTCT – Prevention of mother–to–child transmission

Only two of the included studies involved randomised controlled trials, in both cases with cluster randomisation at the level of the health care facility [36,49]. We furthermore included one non–randomised controlled trial [43], one stepped–wedge cluster non–randomised trial [46], two controlled before–and–after studies [44,48], one prospective [38] (1) and two retrospective [41,42,45] cohort studies, and three historically controlled trials [39,40,47]. For one of the included cluster–RCTs only baseline data were available at the time of the review [36], however, additional data on patient satisfaction with and provider’s perception of the intervention were published separately in a cross–sectional study [35] and as a purely qualitative study [37].

### Description of interventions

Nine of the 12 included studies focused on integration of HIV–related services with ANC. Of these, four studies focused exclusively on integration of antiretroviral therapy (ART) for HIV–infected pregnant women with ANC services [40,41,45,46], four on measures for PMTCT of HIV infection [38,39,44,49], and one on HIV care and treatment services for both mother and child [35–37]. Additionally, two studies discussed the integration of syphilis screening and treatment services with ANC [43,49]. Only one study described the integration of services during the postnatal care period with ANC services [48]. All of the included studies described integration primarily from the perspective of delivery of services. While the necessity for integration of other health system functions was briefly touched upon in the study by Pfeiffer and others [45], this was not described as part of the intervention.

In the included studies, integrated delivery of services generally entailed delivery of multiple services by the same health care provider or by an integrated care team, with all services provided either within the ANC clinic or otherwise within the same premises as the ANC clinic. However, in one study [40], only HIV testing and counselling were fully integrated within the ANC service, whereas initiation and follow–up of treatment for HIV–infected women were performed at a separate facility. In the comparison groups, similar services were usually provided as stand–alone services either within the same facility as the ANC clinic or at a nearby health facility. These services could be accessed by referral from the ANC clinic.

### Quality assessment and risk of bias

Of the included studies, only two met the ‘gold standard’ of evidence offered by the RCT design. All other studies used designs that are generally considered more prone to bias and confounding. The risk of bias for six studies (including the two RCTs, two CBA studies, one NRCT and one stepped–wedge trial) was assessed using the EPOC criteria. Only one of the RCTs described a random method of allocation and reported blinding of the study investigators [36]. The other RCT provided scant methodological detail and the study protocol was not available [49]. Similarly, the two CBA studies [44,48], as well as the NRCT [43] also did not report sufficient methodological information to assess risk of bias. **Table 2** provides a summary of the risk of bias assessment (using EPOC criteria) of included RCTs, SWTs, CBAs and NRCTs.

**Table 2:** Risk of bias assessment (EPOC criteria) of included RCTs, SWTs, CBAs and NRCTs

	Munkhuu 2009 [49] (cRCT)	Turan 2012 [36] (cRCT)	Killam 2010 [46] (SWT)	Geelhoed 2013 [44] (CBA)	Rahman 2011 [48] (CBA)	Bronzan 2007 [43] (NRCT)
Sequence generation	U	L	H	L	N/A	N/A
Allocation concealment	U	U	H	U	N/A	N/A
Blinding	U	L	L	U	U	U
Complete outcome data	L	L	L	N/A	N/A	N/A
No selective outcome reporting	U	N/A	U	U	U	U
Group comparability	L	L	L	U	L	U
Protection against contamination	U	L	U	U	U	H
Free from other sources of bias	L	U	L	U	L	H

cRCT – cluster–randomized controlled trial, CBA – controlled before–and–after trial, NRCT – non–randomised controlled trial, SWT – stepped wedge trial, H – High risk, L – Low risk, U – Unclear, N/A – Not applicable

For the remaining six studies, the risk of bias was rated against the three categories of the Newcastle–Ottawa scale. **Table 3** shows the risk of bias assessment for included NRS based on the Newcastle–Ottawa Scale. For the study group category, one cohort study [35] and two historically controlled trials [40,47] scored the maximum of four stars; two studies (presented in three papers) received three stars [39,41,42] and one study failed to provide information on all but one of these criteria, receiving 1 star [45]. For the group comparability category, no studies received two stars. For the outcome category, five studies [35–39,44] reported the use of routine clinic and programme records to collect data, which may be assumed secure; one study did not report its data source at all [45]. As all included NRS used uptake and utilisation of services during pregnancy as their primary outcome, the period of follow–up until delivery was considered sufficient for all seven studies. This also meant that “loss to follow–up” was not applicable in most cases, as no follow–up beyond the point of recorded uptake of services was required. Hence, we did not award any stars in this category. Overall, one cohort study [35] and one HCT [40] scored seven or eight stars; three studies scored five or six stars [39,41,42,47] and one scored just two stars [45].

**Table 3:** Risk of bias assessment for included NRS based on the Newcastle–Ottawa scale

	Ong’ech 2012 [38]	Pfeiffer 2010 [45]	Stinson 2010, 2013 [41,42]	Kasenga 2009 [47] (HCT)	Van der Merwe 2006 [40] (HCT)	van’t Hoog 2005 [39] (HCT)
**Study group:**						
Representativeness	★	–	★	★	★	★
Selection of control	★	★	–	★	★	★
Exposure	★	–	★	★	★	–
Baseline	★	–	★	★	★	★
**Cohort comparability:**	★	–	★	–	★	–
**Outcome:**						
Assessment methods	★	–	★	★	★	★
Follow–up	★	★	★	★	★	★
Loss–to–follow–up*	–	–	–	–	–	–
**Total**	**7 stars**	**2 stars**	**6 stars**	**6 stars**	**7 stars**	**5 stars**

*As all included NRS used uptake and utilisation of services during pregnancy as their primary outcome, no follow–up beyond the point of recorded uptake of services was reported. We therefore did not award any stars in this category.

### Uptake and utilisation of health services

**Figure 2:**
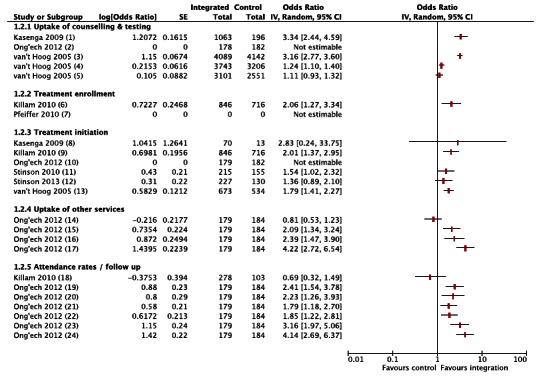
Uptake and utilisation of HIV services (integrated care vs controls). (1) HIV testing within ANC; (2) Infant DBS–PCR testing at 6–8 weeks; (3) Pre–test counselling; (4) Post–test counselling; (5) HIV testing within ANC; (6) Enrollment to HIV–care within 60 days of diagnosis; (7) Women registered for HIV care <30 days post–test (missing data, contact); (8) Nevirapine at delivery; (9) ART initiation during pregnancy; (10) Infant CTX initiation at 6–8 weeks (100% success in intervention group); (11) ART; (12) HAART; (13) Nevirapine uptake; (14) Measles immunization at 9 months; (15) Oral polio vaccine at 14 weeks nths; (16) Complete vaccination by 12 months; (17) DPT vaccine at 14 weeks; (18) 90–day retention among patients initiating ART; (19) 9–month postnatal visit; (20) 6–month postnatal visit; (21) Continuation of CTX prophylaxis at 6 months; (22) 14–week postnatal visit, (23) 12–month postnatal visit; (24) HIV antibody test at 12 months.

Utilisation outcomes for studies that examined integration of HIV services were grouped into four main themes: uptake of counselling & testing, enrolment, treatment initiation and follow–up & attendance. **Figure 2** shows a Forest plot of uptake and utilisation of HIV services (integrated care vs controls) for the included studies.

Three studies reported outcomes related to uptake of testing and counselling [38,39,47], suggesting higher uptake of HIV testing in integrated clinics [39,47]. Treatment initiation was higher in integrated clinics: one of the studies which did not find an effect had a very small sample size [47], and more recent outcomes from the same study as Stinson 2010 reported positive effects [41]. Effect on uptake of services and treatment initiation could not be estimated in Ong’ech and others, as all PCR testing and co–trimaxazole initiation was complete in both intervention and control groups. In the CBA study [44,48], there was an improvement in follow–up of HIV–exposed infants (registration, follow–up visits, serological testing) in both groups, but the progress could not be attributed to integrated MCH services and difference–in–difference estimates were not provided. Only one study reported on uptake of other services (immunisations for HIV infected infants) and follow–up care (attendance at PNC appointments, and continuation with prophylaxis), and suggested that integrated HIV services improved continuity of care for HIV infected infants [38].

For HIV–services, three studies reported on timeliness of treatment initiation or treatment duration at delivery [40,41,46]. Time to receiving test results and time to treatment initiation were shorter in integrated delivery models than in control groups in all three studies. Duration of ART before delivery and gestational age at ART initiation were comparable across integrated and control service delivery models. **Table 4** summarises the findings from the included studies on the timeliness of treatment initiation.

**Table 4:** Timeliness of treatment initiation

	Measure	Integrated	Control	*P*–value
**Duration of ART before delivery (weeks):**				
Killam 2010 [46]	Mean (SD)	10 (N/A)	11 (N/A)	NS
van der Merwe 2006 [40]	Median (IQR)	7 (3.9–11.2)	5 (2–10)	NS
**Gestational age at ART initiation (weeks):**				
Killam 2010 [46]	Mean (SD)	22 (N/A)	22 (N/A)	NS
van der Merwe 2006 [40]	Median (IQR)	32 (28–35)	33.5 (31–36)	0.042
Stinson 2013 [41]	Median (IQR)	31 (28–34)	30 (27–34)	NS
**Time to receiving CD4 cell count (days):**				
van der Merwe 2006 [40]	Median (IQR)	29 (11.5–45)	50 (22–92)	0.047
**Time to treatment initiation (days):**				
Stinson 2013 [41]	Median (IQR)	36 (N/A)	59 (N/A)	<0.001
van der Merwe 2006 [40]	Median (IQR)	37 (22–63)	56 (30–103)	0.041

SD – standard deviation, IQR – interquartile range, N/A – not applicable, NS – not significant

**Figure 3:**
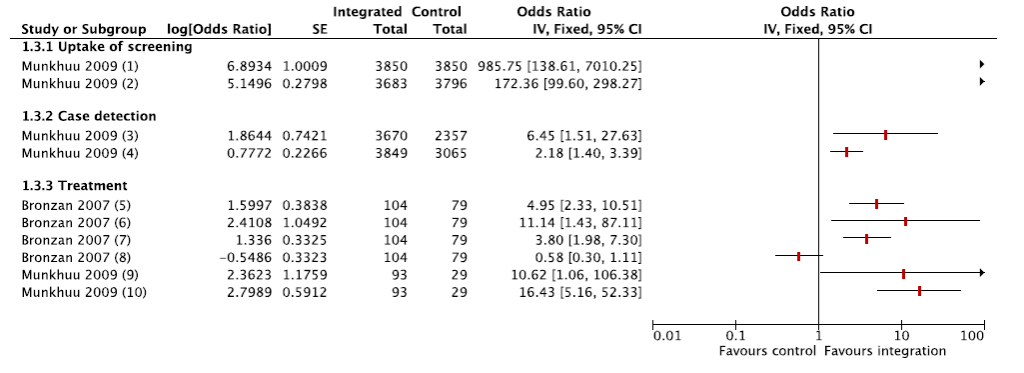
Uptake and utilization of syphilis screening services (integrated care vs controls). (1) Coverage at 1^st^ antenatal visit; (2) Coverage at 3^rd^ trimester; (3) Cases at 1^st^ antenatal visit; (4) Cases at 3^rd^ trimester; (5) At least one appropriately timed penicillin dose/week; (6) One appropriately timed penicillin dose/week; (7) Two appropriately timed penicillin doses/week; (8) Three appropriately timed penicillin doses/week; (9) Adequate treatment; (10) Partner treatment.

Two studies reported uptake and utilisation of services after integration of syphilis screening to ANC services [43,49]. Syphilis screening coverage was universal in the integrated model at the first antenatal visit, and was still significantly higher during the third trimester as compared with the control group; therefore, case detection was also higher in the intervention group. Appropriate treatment for patients with syphilis and their partners also improved in the integrated care delivery models. **Figure 3** shows a Forest plot of the results of uptake and utilization of syphilis screening services (integrated care vs controls) for the included studies.

Only one study reported outcomes relevant to integrating ANC to PNC; however, the study examined a multifaceted service delivery intervention involving strengthening both community and facility based care, as well as implementing evidence–based care [48]. While ANC coverage, facility delivery, and caesarean section rates were significantly higher in the post intervention period, the progress may not be attributable to the intervention.

### Health outcomes

**Figure 4:**
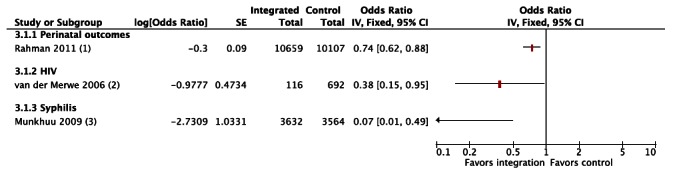
Health outcomes (odds of adverse health outcomes in integrated care vs controls). (1) Perinatal mortality, adjusted; (2) Number of HIV infections among infants born to HIV+ mothers; (3) Number of congenital syphilis cases.

Three studies reported health outcomes (**Figure 4** shows a Forest plot of health outcomes, as measured by odds of adverse health outcomes in integrated care vs controls) [40,48,49]. The results were not pooled due to heterogeneity in type of service integration. One study found that both stillbirths and neonatal deaths were lower in regions where an integrated package of strengthened ANC and PNC services was delivered by community health workers, as compared with usual government care, and the adjusted odds ratio (OR) for perinatal deaths in intervention settings was 0.74 (95% confidence interval (CI) 0.62–0.88) [48]. The numbers of HIV–infected infants born to HIV+ mothers and those with congenital syphilis also were lower where testing and counselling were integrated to ANC services [40,49].

### User experience

Data on user experience with and preferences regarding integrated care were collected in one sub–study of a cluster randomised trial of HIV–integrated services [35,36]. In adjusted models, overall user satisfaction with care was associated with a preference for integrated services (odds ratio, OR = 2.03, 95% CI 1.07–3.85), and attending an integrated clinic (OR 10.34, 95% CI 2.08–51.3). Interactions between HIV status and integration suggest that integration improved HIV–infected women’s satisfaction with their overall clinic experience, while it did not have an effect on HIV–uninfected women [35]. One study reported on the satisfaction of caretakers for HIV–infected infants in intervention and control groups, but did not provide any data [38]. At the end of one year of follow–up, there was no difference in satisfaction with the integrated vs usual care models.

Two studies reported on user satisfaction for the intervention groups only [44,49]. For one–stop integrated MCH services for HIV–infected infants, health care providers reported high satisfaction and “a subjective feeling of increased effectiveness” [44]. Over 86% of women attending two antenatal clinics in Ulaanbaatar, Mongolia, strongly agreed or agreed that they preferred receiving syphilis testing in the same place as ANC, allowing them to get same–day results and receive counselling and treatment from ANC providers. 80% were satisfied with the one–stop service, but 38% found the rapid testing stressful and less confidential. Most providers were also satisfied with integrated services, not reporting any significant problems or that syphilis counselling and treatment interfered with routine antenatal care [49]. Providers report, however, that integrated services lead to high staff workloads [44,49].

## DISCUSSION

We found 12 studies that compared delivery of health services integrated into ANC with other, non–integrated, models of delivery of the same set of services. Our review finds some, albeit limited, evidence that integrated delivery results in improved uptake and utilisation of these services. Increased uptake of testing (HIV and syphilis) and PMTCT services, and earlier initiation of ART for HIV–infected mothers were, in turn, associated with lower rates of congenital infection with HIV and syphilis. In general, women also reported improved satisfaction with integrated services. These findings support the view that integrating additional health services into ANC can result in improved access to and uptake of essential health services for pregnant women. However, the reported evidence is largely based on non–randomised studies with moderate– to high–risk of bias, and therefore should be interpreted with caution.

### Overall completeness and applicability of evidence

This review adds to a growing body of literature on integration of specific services into antenatal care settings, such as PMTCT [11,19,26] and HIV services [25]. Of special interest is the review by Tudor Car [24], which looks at the effect of integration of perinatal PMTCT interventions aimed at reducing MTCT of HIV. It bases its findings on five studies, of which four were included in this review. It found that “there is very limited, non–generalisable evidence of improved PMTCT intervention uptake in integrated PMTCT programmes.” A separate review by Lindegren and others looked at the impact of integrating HIV services with Maternal, Neonatal and Child Health (MNCH) services [25]. The focus of the review by Lindgren and others different from ours in that it looked at integration of HIV services into ANC, but also considered the reverse (ie, integration of ANC services into HIV services), or integration of both types of services into a pre–existing set of services. Across these different forms of integration, Lindegren and others found that for most studies integration had an apparent positive impact on reported outcomes. Several studies included in the review by Lindegren and others reported mixed or no effects, and one study reported negative outcomes due to providing integrated services [25]. These findings are generally consistent with those reported in our review.

Strikingly, the large majority of studies (nine out of 12) we retrieved concerned the integration of HIV–related services, in particular PMTCT and ART, into ANC. Two other studies dealt with integration of syphilis screening into ANC. However, we found no studies on integration of, for example, screening and treatment for other STIs, tuberculosis, malaria, non–communicable diseases or mental health issues into ANC that met the inclusion criteria. Whilst this emphasis on HIV is perhaps understandable in the context of countries with a high burden of HIV, this review reveals that there are few studies that have explored the potential of using ANC contacts as an entry point for health care services for women. This apparent deficiency was previously also addressed by Kerber and others, who noted that even in countries with good coverage of ANC services, coverage of effective interventions such as PMTCT remains low [5]. Since ANC often represents the most important, if not the only, point of contact a woman in LMIC has with formal health care services, our findings demonstrate lost opportunities for providing essential preventive and curative services.

Furthermore, the almost complete absence of studies looking at the potential benefits of integrating PNC services with ANC underscores the insufficient attention given to PNC in general, and suggests continued fragmentation of the continuum of maternal and child health care, particularly in the crucial post–partum period. As Kerber and others remarked, this fragmentation of the continuum suggests a “consensus has not been reached on a minimum package of postnatal interventions, with the strategies and mix of skills that are necessary for delivery.” [5] This is a critical shortcoming that urgently needs to be addressed.

Only two of the included studies explicitly addressed the potential drawbacks of service integration and its impact on service quality, noting that integrated delivery of services could theoretically lead to inadvertent disclosure of HIV status as HIV–infected women would require longer appointments than non–infected women [37], and could result in unnecessary treatment if the new service model requires easier–to–use but less accurate testing techniques [49]. One study found that nurses considered the impact of integration on their workload acceptable [44]; no other impacts on the health system or other health services were discussed. This limited attention to the impact of integration on service quality and on the wider health system is cause for some concern. Decisions on whether or not to integrate specific services should be based on system–wide consideration of *all* potential costs and consequences, including unintended ones. However, the studies included in the review did not estimate costs and economic consequences of integration.

### Potential biases and limitations

This review has four main limitations. First, although we used a robust and tested search strategy, it is nonetheless possible that we missed relevant studies. However, comparison with other reviews with a similar scope (ie, integration of services into maternal and child health care) [11,24,25,50], validates our strategy as we retrieved all relevant titles cited there.

Second, we were unable to retrieve the full text for 23 publications that we considered potentially eligible based on their titles and, where available, abstracts. Many of these were published in national or regional journals, often in languages other than English. Whilst this may have skewed our findings towards studies set in Anglophone countries and those published by European and North American researchers, it should be noted that out of the 23 missing studies only three were published from 2000 onwards. By comparison, all included studies were published in 2005 or later. We therefore consider it unlikely that many of the missing studies would have been eligible for inclusion, or that this could have had a significant effect on our overall findings.

Third, a potentially more important source of information not reported here is formed by programme evaluations that have not been published in the peer–reviewed literature, but have been prepared by funding institutions and implementing organisations. Such additional data will be included in a supplementary paper, which more generally discusses barriers and enablers to integration of services into ANC [51].

Fourth, as our review focused specifically on the impact of a service delivery model in which services were integrated into ANC, we required studies to compare findings to a service model in which the same, or a similar, set of services was provided in a non–integrated fashion. Without such a comparison it would not have been possible to distinguish between outcomes due to the availability of the services themselves, and those related to their mode of delivery. As a result, we excluded studies in which services that had not been previously available were directly introduced into the ANC setting. This applied in particular to PMTCT services. Also studies that did not clearly describe whether services had been previously available or, if so, how these were delivered, had to be excluded. This limited our evidence base to studies that very explicitly compared service delivery models, despite the fact that others also discussed similar integrated services.

## CONCLUSIONS

### Implications for policy and practice

This review highlights the potential for improving maternal and child health care by integrating additional services with antenatal care, capitalising on the opportunities presented by relatively high rates of ANC coverage in many LMICs to develop integrated, evidence–based and cost–effective interventions with common delivery strategies for target populations [5]. The content and complexity of such a service package should be informed by the local health system capacity and epidemiological context and can evolve over time. However, care should also be taken to minimise the risks involved, such as potential deterioration of service quality and patient satisfaction, or overburdening frontline health workers.

### Implications for research

There is a large evidence gap on the possible impacts for uptake and utilisation of essential services and health outcomes from integration of services with ANC. What little evidence is available is of insufficient quality to allow formulation of policy recommendations for other LMICs that may benefit from integration of health services. There is a clear need for more rigorously conducted studies, ideally involving comparison between different service delivery models with random allocation. However, additional quasi–experimental studies, and demonstration projects complemented by modelling studies, could also provide valuable insights in this area and in particular should help in understanding the role of contextual factors in achieving specific outcomes.
